# Management of patients with adrenal myelolipoma: experience from a tertiary referral centre

**DOI:** 10.1007/s11845-024-03779-2

**Published:** 2024-09-04

**Authors:** Anant Paul, Conor Toale, Marie Egan, Maria Whelan, John Feeney, Stephen Crowther, James Gibney, Kevin Conlon

**Affiliations:** 1 Department of Surgery, Tallaght University Hospital, Dublin, Ireland; 2 Department of Endocrinology, Tallaght University Hospital, Dublin, Ireland; 3 Department of Radiology, Tallaght University Hospital, Dublin, Ireland; 4 Department of Histopathology, Tallaght University Hospital, Dublin, Ireland; 5 School of Medicine, Trinity College Dublin, The University of Dublin, Dublin, Ireland; 6 Trinity Centre for Health Sciences at Tallaght University Hospital, Dublin 24, Dublin, Ireland

**Keywords:** Adrenal, Adrenal surgery, Adrenal surveillance, Adrenalectomy, Incidentalomas, Myelolipoma

## Abstract

**Background:**

Adrenal myelolipomas are rare, benign, tumours of the adrenal cortex.

**Aims:**

This study reports the experience of a tertiary adrenal surgery referral centre’s approach to the management of patients with adrenal myelolipoma.

**Methods:**

A retrospective observational cohort study was conducted on all adult patients (> 18 years age) diagnosed with adrenal myelolipoma from January 1, 2014, to December 30, 2022. Demographics, imaging characteristics, histological diagnosis (where applicable) and follow-up data were compared between patients undergoing surgery and those referred to surveillance. Indications for operative intervention were recorded at the time of multidisciplinary team discussion, consisting of surgeons, endocrinology physicians, radiologists, pathologists and specialist nursing representatives.

**Results:**

Of the 522 patients with an adrenal lesion discussed in adrenal tumour meeting between 2014 and 2022, *n* = 15 (2.8%) were diagnosed with adrenal myelolipoma. Of the 15 patients, 4 underwent adrenalectomy at first presentation (27%), while 1 patient underwent adrenalectomy after interval follow-up. Indications for operative intervention were as follows: ‘indeterminate lesion’ (*n* = 3), ‘abdominal pain and size (> 4 cm)’ (*n* = 1) and ‘mass effect on adjacent organs’ (*n* = 1). The mean rate of lesion growth in patients referred for surveillance (*n* = 10) was 0.13 cm/year. Histology confirmed adrenal myelolipoma as the diagnosis in all resected tumours.

**Conclusions:**

For patients with adrenal myelolipoma, the presence of symptoms and/or indeterminate features on imaging may be more clinically useful indications for operative intervention over size alone. The surveillance of adrenal myelolipomas, even in patients with adrenal lesions > 4 cm, is a safe clinical strategy, provided the imaging characteristics are benign and patients remain asymptomatic.

## Introduction

Adrenal myelolipoma are rare, benign, mesenchymal and stromal tumours of the adrenal cortex composed of a variable proportion of mature fat and hematopoietic tissues [1]. They are diagnosed in 1 out of every 500–1250 autopsy cases, account for 6–16% of adrenal incidentalomas (AI) and are the second most common tumour originating from adrenal cortex [2–4]. The lesions appear to affect both sexes equally and are most often diagnosed between fifth and seventh decades of life [5]. Adrenal myelolipomas which are greater than 6 cm are considered large, while those greater than 10 cm are considered ‘giant’ [4–6]. Predominantly slow-growing and unilateral, adrenal myelolipomas are asymptomatic in 95% of cases, though reported symptoms include early satiety, abdominal fullness, abdominal discomfort, back and flank pain and positional shortness of breath [7]. The rate of growth size over which these are likely to become symptomatic is unknown, and our understanding of progression is based on a small number of retrospective studies [7–9].

A prior study by Shenoy et al. (2015) demonstrated a 75% increase in cases of adrenal myelolipoma diagnoses since the year 2000, thought to be a result of increasing usage of abdominal imaging [10].

Despite this, underlying triggers of growth are not well understood with pathological stimuli from trauma, infection or long-term ACTH stimulation being postulated as possible mechanism [10–12].

There is a further paucity in understanding regarding the natural history and progression of this benign entity. The increasing rate of incidental identification of these tumours leads to the clinical question of whether these presumed benign tumours require surgical intervention at time of initial diagnosis and for what indications or whether they require surveillance [8, 13]. Commonly cited indications for the surgical management of adrenal myelolipomas are the presence of symptoms, > 4 cm size, atypical radiological appearance, evidence of mass effect, a suspected functional tumour, haemorrhage and rupture [5, 14–17]. However, evidence underpinning the rationale for these indications is largely based on single-institution retrospective studies and case series [5, 7, 18–20].

Our institution is a tertiary referral centre for adrenal surgery with a formalised management pathway based on outcomes from an adrenal multidisciplinary tumour board meeting. With this study, we aimed to analyse our institution’s approach to management of adrenal myelolipoma and compare to international published literature. We report the rate of up-front operative intervention and indications for surgery as decided by a multidisciplinary team of surgeons, radiologists and endocrinology physicians. We further report the rate of lesion growth and rate of operative intervention in patients initially referred for surveillance. We also aimed to investigate any observed difference in mean growth rate within the unresected adrenal myelolipomas during surveillance, when comparing groups-based size at initial diagnosis (< 4 cm and > 4 cm) in size. These findings were used to evaluate the rationale for surveillance in the patient population with unresected adrenal myelolipoma.

## Methods

An observational cohort study was conducted on all adult patient (> 18 years age) diagnosed with adrenal myelolipoma at our institution, by retrospectively interrogating a prospectively maintained database. We chose the study period between 1st January 2014 to 30th December 2022 as the actual number of adrenal lesions being referred to our centre was registered onto the database from this date, with the introduction of adrenal multidisciplinary tumour board pathway within our institution. The adrenal multidisciplinary tumour board consisted of a dedicated surgeon performing high volume adrenal cases, radiologists experienced in adrenal reporting, endocrinologists and speciality pathologist. All patients with a primary radiological diagnosis of adrenal myelolipoma were eligible for inclusion. Patients who underwent adrenal surgery for any another indication and had an incidental finding of adrenal myelolipoma as second pathology on histology were not eligible for inclusion. The surgery group comprised of patients who had adrenalectomy and surveillance group formed of patients undergoing follow-up with interval imaging and clinical follow-up. Charts and discussion notes from adrenal multidisciplinary tumour board meetings were reviewed for this time period. Patient demographics were recorded, along with documented symptoms and relevant comorbidities at time of initial diagnosis. The imaging confirmed adrenal myelolipomas and indeterminate adrenal lesions characterised on preoperative imaging with computed tomography (CT) or magnetic resonance imaging (MRI) including size, side and location, and Hounsfield units were recorded along with results of biochemical workup. In patients within the surveillance group, the following variables were recorded during follow-up: change in characteristic or size of adrenal myelolipoma, duration of follow-up, any new onset of symptoms, onset of complications and number of repeat adrenal multidisciplinary tumour board discussions.

### Data analysis

Continuous data are presented as mean ± standard deviation (SD) and categorical data presented as *n* (%). Means were compared using Student *t*-tests. Correlations between continuous variables were analysed using Pearson correlation coefficients. A *p*-value of < 0.05 was considered statistically significant. Statistical Package for the Social Sciences (SPSS) statistical software (version 28.0 IBM) was used for data analysis. Institutional approval was provided by Tallaght University Hospital.

## Results

**Fig. 1:**
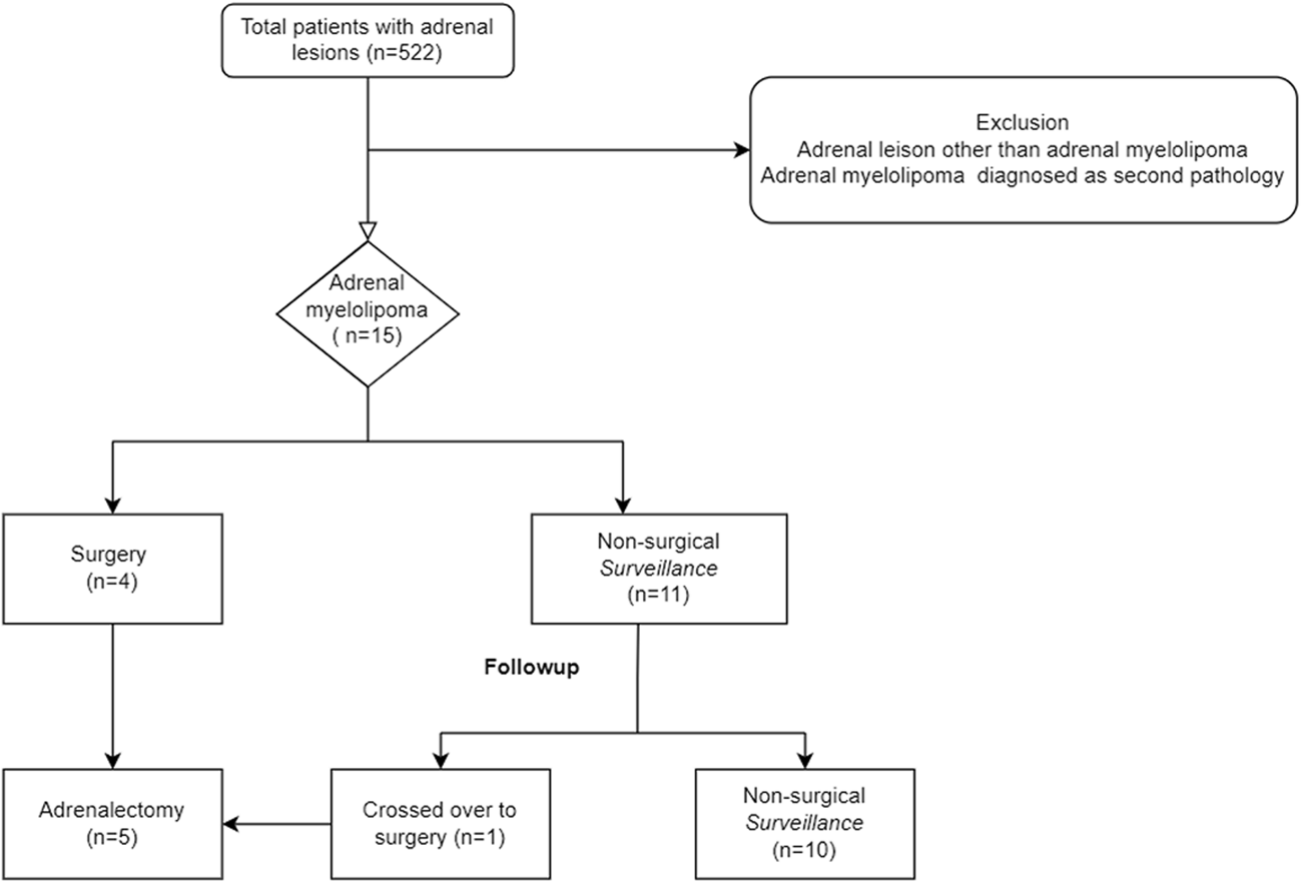
Management pathway for patients diagnosed with adrenal myelolipoma in our institution

**Fig. 2:**
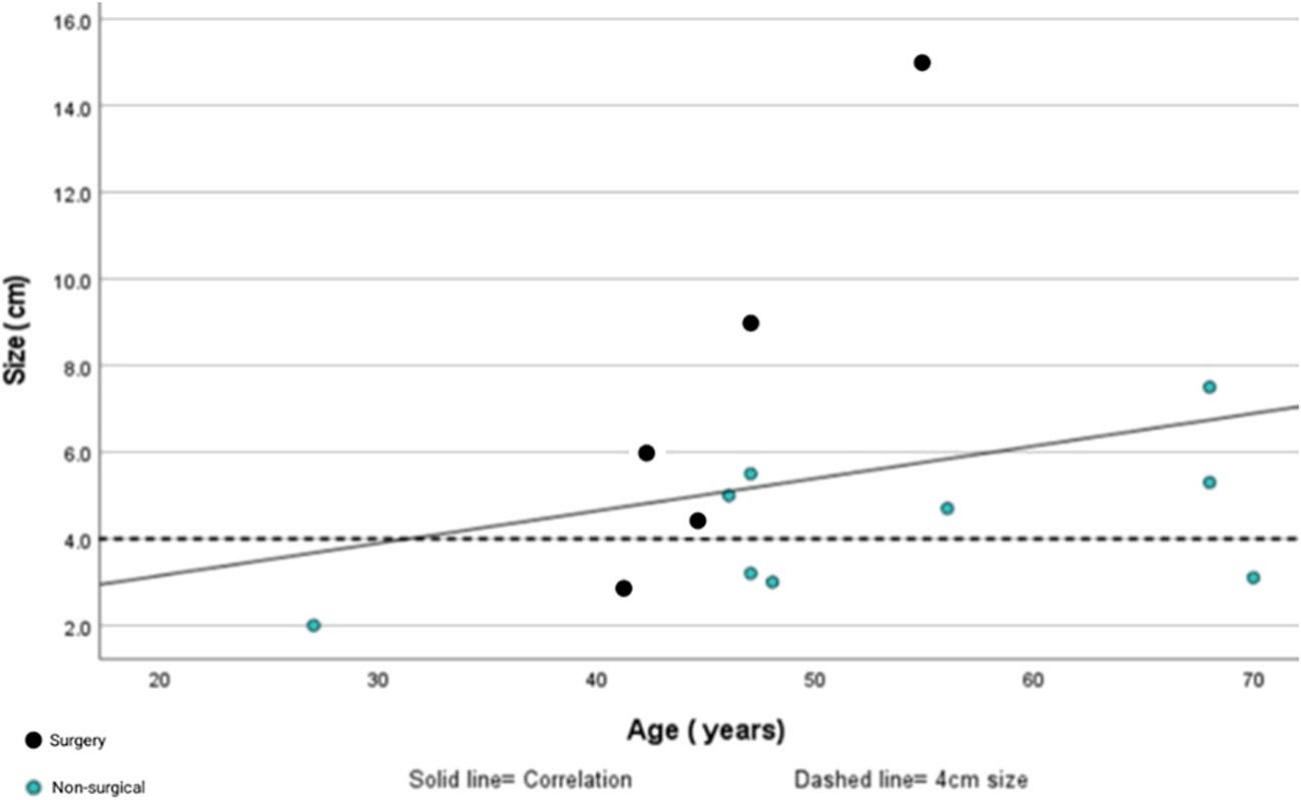
Correlation between age at diagnosis and size (cm) in adrenal myelolipoma

Of the 522, patients with an adrenal lesion discussed in adrenal tumour board meeting between 2014 and 2022, *n* = 15 (2.8%), were diagnosed with adrenal myelolipoma with radiological imaging or on histology for indeterminate adrenal lesions (Fig. 1). All patients reported abdominal symptoms, though 12 patients had other intra-abdominal pathology that could have accounted for their symptoms. Patients ranged in age from 27 to 70 years with mean age (SD) of 50 (11.5) years and with a male-to-female ratio of 2:3. The mean (SD) age of patients within surgery group was 47 (4.8) years, and the surveillance group was 51 (13.1) years. The mean (SD) size of identified adrenal myelolipomas was 5.4 (3.2) cm at diagnosis. Table 1 summarises the demographics, radiological and clinical characteristics of the surgery and surveillance groups. Of the 15 patients, 4 underwent adrenalectomy at first presentation (27%), while 1 patient underwent adrenalectomy after crossing over from surveillance group. Ten patients within surveillance group continued to have follow-up (Fig. 1). There was no significant relationship found between the age of diagnosis and the size of adrenal myelolipoma at initial diagnosis on statistical analysis (*r* = 0.26, *p* = 0.33) (Fig. 2).

**Table 1:** A demographic and clinical comparison of ‘surgery’ and ‘surveillance’ groups for patients with adrenal myelolipoma presenting to our institution

	Surgery (*n* = 5)	Surveillance (*n* = 10)	*p*-value
Age [mean (SD)]	47 (4.8)	51 (13.1)	0.418*
Gender *n* (%)			1.00+
▪ Male	2 (13.3%)	4 (26.7%)	
▪ Female	3 (20%)	6 (40%)	
Tumour characteristics Size (cm) [mean (SD)]	7.4 (4.7)	4.3 (1.5)	0.221*
Size (cm)			0.600 +
• > 4 cm *n* (%)	4 (26.7%)	6 (40%)	
• < 4 cm *n* (%)	1 (6.6%)	4 (26.7%)	
Radiology imaging: confirmed AML	2	10	
Radiology imaging: indeterminate lesion, primary diagnosis of adrenal myelolipoma	3	0	
Hormonal active/inactive *n* (%)	0/5 (33.3%)	0/10 (66.7%)	
Location *n* (%)			
• Right	4 (26.6%)	7 (46.7%)	
• Left	1 (6.6%)	3 (20%)	
• Bilateral	0	0	
Clinical characteristics *n* (%)			
▪ Asymptomatic	2 (13.3%)	10 (66.7%)	0.022 +
• Abdominal pain & discomfort	3 (20%)	0	

Data are presented as number (%) or means + / − SD (standard deviation). Statistical analyses were performed using independent *t*-test* and Fisher exact test + . *p*-value < 0.05 considered significant

### Surgery group

Of the five patients undergoing surgery, the documented indications for operative intervention were as follows: ‘indeterminate lesion’ (*n* = 2), ‘abdominal pain and size’ (*n* = 1) and ‘mass effect on adjacent organs’ (*n* = 1). One further patient crossed over from surveillance towards surgery after 1 year of follow-up due to new onset of abdominal pain and subsequent repeat imaging findings suggestive of an ‘indeterminate’ lesion. The histology of all resected adrenal tumours confirmed adrenal myelolipoma in all five pathological specimens. On follow-up, patients with symptoms attributable to their adrenal reported complete resolution following surgery.

### Surveillance group

Of the *n* = 10 patients within the surveillance group, *n* = 8 have since undergone interval radiological imaging. Two recently diagnosed patients are yet to undergo repeat imaging. A change in size of the adrenal myelolipoma was observed in seven patients with no change in size in one patient. The duration of interval imaging follow-up ranged between 1 and 18 years. Repeat imaging varied between 1 and 5 scans depending on the duration of follow-up. A mean growth rate of 0.13 cm/year was observed. On further analysis, adrenal myelolipomas with initial size of > 4 cm (*N* = 6) showed a mean (SD) growth per year of 0.20 (0.12) cm, and those of < 4 cm (*N* = 4) showed a mean (SD) growth per year of 0.11 (0.16) cm (*p* = 0.421). In one case, the tumour grew by 6.5 cm after 11-year follow-up which on discussion on adrenal multidisciplinary tumour board meeting was felt to be due to bleed into the adrenal gland secondary to a trauma. As patient was stable and asymptomatic, conservative approach was recommended after MDT, and subsequent interval imaging demonstrated significant reduction in the adrenal tumour size. One patient who was observed over 9 years did not develop any change in size. The number of adrenal multidisciplinary tumour board discussions varied from range of 1 to 8 depending on the change noted on the interval radiology imaging.

## Discussion

This study reports the management of 15 patients with adrenal myelolipomas at a tertiary adrenal surgery referral centre. Indications for up-front surgery were indeterminate features on radiology (*n* = 2), attributable symptoms (*n* = 1) and mass effect on surrounding organs (*n* = 1). No patient underwent surgery based on the size of the lesion alone. Of the 11 patients undergoing surveillance, only 1 subsequently underwent surgery due to the development of indeterminate radiological features on subsequent imaging with new onset abdominal symptoms. Adrenal myelolipoma was the final histological diagnosis in all cases. In all patients undergoing surveillance, the rate of growth was low at 0.13 cm/year, including 6 of 10 patients with lesions > 4 cm on initial investigation. These findings suggest that surveillance for presumed myelolipomas in the absence of concerning radiological features or symptoms is safe strategy.

In our cohort, adrenal myelolipoma comprised of 2.8% of total cases of adrenal lesions. This incidence is lower than the literature with case series reporting incidence between 4 and 10% [7, 21–23]. This may reflect a referral bias that not all cases of adrenal myelolipoma have been reviewed at the adrenal multidisciplinary tumour board. The mean age for diagnosis was between fourth to fifth decade with range of age at initial diagnosis from 27 to 70 years, with female predominance (Table 1). This has been similar to reported cases in different studies [7, 24–26]. The size of the adrenal lesions ranged from 2 to 15 cm, comparable to the reported literature [14, 27]. All resected adrenal lesions within this cohort were benign.

There is a gap in understanding the true progression of these tumours which influences the decisions in management of adrenal myelolipoma. The recommendations based on size are encompassed under the guidelines of benign adrenal lesions (< 4 cm and > 4 cm) as per the European Society of Endocrinology clinical practice guidelines (ESE/ENSAT) 2016 [28]. In our prospective series representing one of the largest single-centre experiences, > 65% adrenal myelolipoma tumours were > 4 cm: surgery group mean size was 7.4 cm (4.7), and surveillance group was 4.3 cm (1.5) (Table 1). Although the surgery group mean size was greater compared to surveillance group, almost 40% of patients within surveillance group in comparison with 26% patients in surgery group had adrenal myelolipoma size of > 4 cm (Fig. 2). This shows that despite the mean difference in size between both groups, size was not the only independent factor in the decision process.

### Surgery in adrenal myelolipoma

Indications for surgery in adrenal myelolipoma are based largely on retrospective studies of case-based reports and expert opinions. *Gershuni *et al*. (2014)* in a review of 16 patients with adrenal myelolipomas noted the following indications for adrenalectomy: abdominal pain (44%), large tumour > 8 cm (50%), atypical radiological appearance (31%) and inferior vena cava compression (7%) [14]. *Shenoy *et al*. (2015)* in retrospective review of 584 cases recommended the following indications for surgery: symptomatic tumour, size > 4 cm, metabolically active tumour and suspicion of malignancy on imaging study [10]. *Weber *et al*. (2019)* reported 12 patients with adrenal myelolipoma who underwent surgery and categorised them into 3 clinical groups: the presence of local symptoms, radiological suspicious sign or the presence of local symptom and radiological suspicious sign [17]. *Calissendorff *et al*. (2021)* recommended option of surgery for large adrenal myelolipoma (> 6 cm) or indeterminate lesion and definitive surgery for mass effect or acute haemorrhage within the adrenal myelolipoma [5]. In our study (*n* = 15), the indications for surgery were for radiologically indeterminate lesions (*n* = 2), symptomatic and indeterminate lesions (*n* = 1), symptomatic lesions with a change in size (*n* = 1) and mass effect on surrounding organs (*n* = 1). These decision factors are consistent with the reported literature.

Hamidi et al. in their large case series of 321 patients suggested adrenal myelolipomas > 6 cm in size are more symptomatic [7]. *Han *et al*. (1997)* reported that nearly 50% of those from observed group either had an increase in size or became symptomatic [8]. In our study, majority of the patients remained asymptomatic despite the growth in size of adrenal myelolipoma. Within our surgery group, three patients with symptoms had adrenal myelolipoma measuring > 6 cm. One of them was a case of giant adrenal myelolipoma measuring 15 cm causing mass effect on intra-abdominal adjoining organs. However, within surveillance group, one patient with a lesion < 4 cm became symptomatic and indeterminate on imaging, while one patient with size > 6 cm remained asymptomatic. There was no statistical significance found on correlation between age of diagnosis and size of the adrenal myelolipoma (Fig. 2). This suggests that symptoms and age of diagnosis are likely independent factors, which need to be taken into consideration along with size to make individualised decisions for surgery versus surveillance.

Indeterminate adrenal lesions pose a challenging dilemma in management. ESE/ENSAT recommends three options for indeterminate lesions in adrenal incidentalomas (AI): immediate additional imaging with different modality, interval imaging with 6–12 months or surgery [28]. *Calissendorrff *et al*.* recommend considering interval imaging or surgery depending on the clinical, biochemical and differential diagnosis parameters when considering options in indeterminate lesions [5]. *Melck *et al*.* from their case series reported 7 of the 16 patients in the observed group for indeterminate lesions crossed over to surgery at mean of 13.1 months [29]. In our study to consider an adrenal lesion indeterminate, the patient underwent combination of different imaging modalities: computed tomography (CT), magnetic resonance imaging (MRI), positron emission tomography (PET-CT) and iodine-131-metaiodobenzylguanidine (MIBG) scan. Out of them, three patients had indeterminate lesion in imaging workup for adrenal myelolipoma. After adrenal multidisciplinary tumour board discussion, all three underwent adrenalectomy, based on rationale that indeterminate lesions can masquerade and harbour otherwise ambiguous adrenal pathologies [30, 31]. Adrenal myelolipoma was confirmed on pathology specimen in all three cases. Our study supports surgical intervention in indeterminate lesions as suggested as one of the options by the ESE/ESNAT guidelines [28].

### Surveillance in adrenal myelolipoma

Within the surveillance group with radiologically confirmed adrenal myelolipoma, further decision of surveillance versus no surveillance remains a matter of discussion. Adrenal myelolipomas grow in an indolent manner and remain asymptomatic in majority of cases. Within the surveillance group, our study showed that tumours are slow growing at growth rate of 0.13 cm/year (size range 0.2 to 1.9 cm/year’s range 3 to 18), and in majority of patients, this growth did not trigger move to surgery on adrenal multidisciplinary tumour board discussion. Campbell et al. (2017) in their case series consisting of 69 patients showed that during mean interval surveillance of 3.9 years, 16 tumours showed average growth rate of 0.16 cm/year (0.08–0.71 cm/year). On further analysis of our surveillance group, adrenal myelolipomas with initial size of > 4 cm showed mean growth rate (SD) cm per year by 0.20 (0.12) cm, and those of < 4 cm showed mean growth rate (SD) cm per year by 0.11 (0.16) cm (*p* = 0.421). This would suggest that adrenal myelolipoma measuring > 4 cm tends to grow at faster rate compared to those < 4 cm; however, the underlying factors driving this unpredictable growth pattern may have confounded to the statistical insignificant result. *Campbell *et al*. (2017)* showed association with both younger age and longer duration of time interval between CT scan (mean age: growth group 50 years and no-growth group 62 years) [9]. This would suggest that these tumours if observed over a longer period will grow gradually; however, in majority, cases will still remain within insignificant size to cause any symptoms. In our study, depending on their period of surveillance (1–18 years), patients underwent between 1 and 5 computed tomography interval scans. As the majority of these patients did not need surgical intervention, the potential benefit of repeated interval imaging should be balanced against the radiation exposure from computed tomography imaging.

In rare instances, these tumours can show abrupt growth pattern. Within our study, one patient on radiology interval imaging showed change in size from 5.5 to 12 cm after 11 years of follow-up. After 4 years of interval imaging, the tumour size reduced to 7.3 cm, and patient remained asymptomatic. *Weber *et al*. (2019)* reported change in one patient followed for over 30 years showing increase of 0.15 cm/year from 45- to 92-mm size, while three patients showed > 5-mm growth within 6 months of follow-up [17]. This demonstrates that in exceptional cases, these tumours can exhibit unpredictable growth pattern; however, the majority show slow linear growth. The underlying growth triggers causing sudden changes in size are poorly understood, and over a long duration of follow-up, very few cases will show significant abrupt change in size.

### Limitations

The current study was retrospective as it would be difficult to obtain a prospective study given its rare and clinical course of adrenal myelolipoma. Due to its low prevalence, the true statistical interpretation is limited to show causality or correlation to various factors triggering the growth pattern. There is also gap in understanding the true interval and duration of radiological imaging within the surveillance group.

## Conclusions

Adrenal myelolipomas are benign slow-growing tumours with majority remaining asymptomatic in their clinical course. The decision to proceed to operative intervention should be made by multidisciplinary service, accounting for the presence of symptoms, radiological appearance and patient-specific considerations. The 4-cm size cut-off for surveillance may have less relevance for adrenal lesions showing a typical appearance of an adrenal myelolipoma on imaging and likely should not be considered as the only relevant decision-making factor for surgery. Symptoms and indeterminate radiological imaging-based factors are important to identify. In cases where surveillance is pursued, despite variability in growth pattern, the majority of adrenal myelolipomas remain asymptomatic. Within this asymptomatic group with unequivocal radiology suggesting adrenal myelolipoma, clinical surveillance without interval radiology imaging even for adrenal lesions > 4 cm is a safe clinical strategy. To further strengthen the recommendations for surgery versus surveillance management in adrenal myelolipoma, more prospective data across multicentre adrenal tumour boards will need to be collated to study the true effect of surgery versus surveillance for patients diagnosed with adrenal myelolipoma.

## Data Availability

Aggregated, anonymised data is available upon request.
